# Egg Hatching, Peptide Pheromones, and Endoproteinases in Barnacles

**DOI:** 10.3390/ijms262311393

**Published:** 2025-11-25

**Authors:** Desa Bolger, Joshua Osterberg, Beatriz Orihuela, Arthur Moseley, Daniel Rittschof

**Affiliations:** Duke University Marine Laboratory, Nicholas School of the Environment, 135 Duke Marine Lab Road, Beaufort, NC 28516, USA; joshua.osterberg@duke.edu (J.O.);

**Keywords:** endoproteinase, microbiomes, egg hatching, egg brooding, barnacles, proteomics, exoproteinases

## Abstract

The striped barnacle, *Amphibalanus amphitrite*, is a simultaneous hermaphrodite crustacean that broods eggs. The eggs are physically and enzymatically cleaned in the mantle by the barnacle to manage biofouling during incubation. There is no physiological connection between the embryos and the adult. Instead, barnacles use enzyme products as pheromones to coordinate behavioral, physiological, and biochemical processes involved in egg hatching and larval release. Known larval release pheromones are peptides generated by exogenous trypsins that act on proteins. We characterized barnacle brooding endoproteinases using a proteomic analysis of peptides generated from the hydrolysis of pure proteins that were identified by high-resolution LC electrospray MS/MS. Utilizing pure proteins permitted us to completely identify sequences around proteolytic cleavage sites. Enzyme activity was 2.22 to 2.79 times greater in barnacle and barnacle microbiome samples compared to seawater samples. Distinct enzyme patterns emerged, with higher proline- and asparagine-cutting enzymes in barnacle samples and greater proportions of elastase in seawater. There are at least 13 endoproteinases based on the C-terminus amino acids of peptides, with major contributions from serine proteases. This approach has the potential to provide exceptionally detailed information on endoproteinases in any microbiome assemblage. With a little thought, this technique can be expanded to include exoproteinases as well.

## 1. Introduction

Utilizing “-omics” and more specifically, proteomics, is a way to better understand exogenous proteolytic enzymes and peptides that are generated within organismal responses. Proteinases are among the most ancient of enzymes and have a multitude of functions, including the recycling of ubiquitous amino acid polymers [1]. Proteins in the form of enzymes and structural components of organisms are among the most common biopolymers in animals. As protein construction is a high-energy process, recycling is advantageous if the amino acid building blocks can remain intact for reuse. Protein degradation is accomplished by two basic kinds of proteinases: endoproteinases and exoproteinases. Exoproteinases cleave the terminal amino acid off of peptide chains. Therefore, they are very active when there are a lot of ends of proteins to attack. Endoproteinases cleave proteins within the chain and make new ends. Endoproteinases likely evolved because there was a selective advantage in recycling and generating more ends than there was in creating chains from scratch. However, because recycling is an exogenous operation, a consequence of the activity of the two kinds of proteinases is the generation of free amino acids. These free amino acids can serve as food signals because specific high-information-content peptides can act as identification to initiate for distinct processes [2]. Peptide chemical signals are crucial in the organization of marine communities [2,3]. The use of peptides as signals in marine systems is common and has been reported in echinoderms [4,5], cnidarians [6], mollusks [7,8] and arthropods [9,10]. Proteolytic enzymes that generate peptides from the proteins on animal surfaces are pervasive and contribute to animal body odors [3,8]. Peptide signals and pheromones have short half-lives, large signal-to-noise ratios, large solubility potentials [11], and high information content [2,3]. This information is transmitted at nanomolar to picomolar concentrations [2]. A consequence of this high potency is that the identification of native peptides may be below the detection limits of current analytical chemistry techniques. Previously, demonstration of the nature of the peptides relied on the use of short synthetic mimics and biological assays [2,9,12,13].

Crustaceans use proteases both to manage biofouling on their eggs by cutting the glues that the biofouling organisms use to attach to surfaces [14] and to also identify biofouling on sediments to be used as a food source [15]. Larval-release pheromones in all egg-brooding crustaceans studied involve peptides generated by the action of serine proteases [8]. As peptides are generated by secreted proteases, it is unclear if the pheromones and signals are due to enzymes secreted by the hosts or their microbiome symbionts.

The aim of this project was to better understand how peptides in marine systems are generated and to specifically identify the endoproteinases involved. For the experiments and techniques we report here, we chose striped barnacles (*Amphibalanus amphitrite*) for which peptide analysis during egg hatching has not been conducted. The striped barnacle is a global fouling organism [16,17,18] and is hermaphroditic [19]. Barnacles are small, easy to obtain, and brood eggs with developing embryos. Barnacles are also already known to have peptide body odors in other contexts, as demonstrated by their ability to attract predatory snails and induce larval barnacle settlement [20]. Their eggs are brooded in the mantle instead of being attached to pleopods, as they are in crabs and some shrimp [21]. Peptide pheromones similar to leucocyte chemoattractants synchronize both the embryos and the adults [2,22].

Here, we characterize the endoproteinases released by barnacles and their associated microbiomes by analyzing the peptide products of known pure proteins that we provided as incubates. We incubated barnacles in seawater solutions containing pure proteins, subjected the peptides generated to shotgun proteomics, and then analyzed the peptides generated from the pure proteins to deduce the kinds of proteases active in the host and microbiome community.

## 2. Results

**Figure 1:**
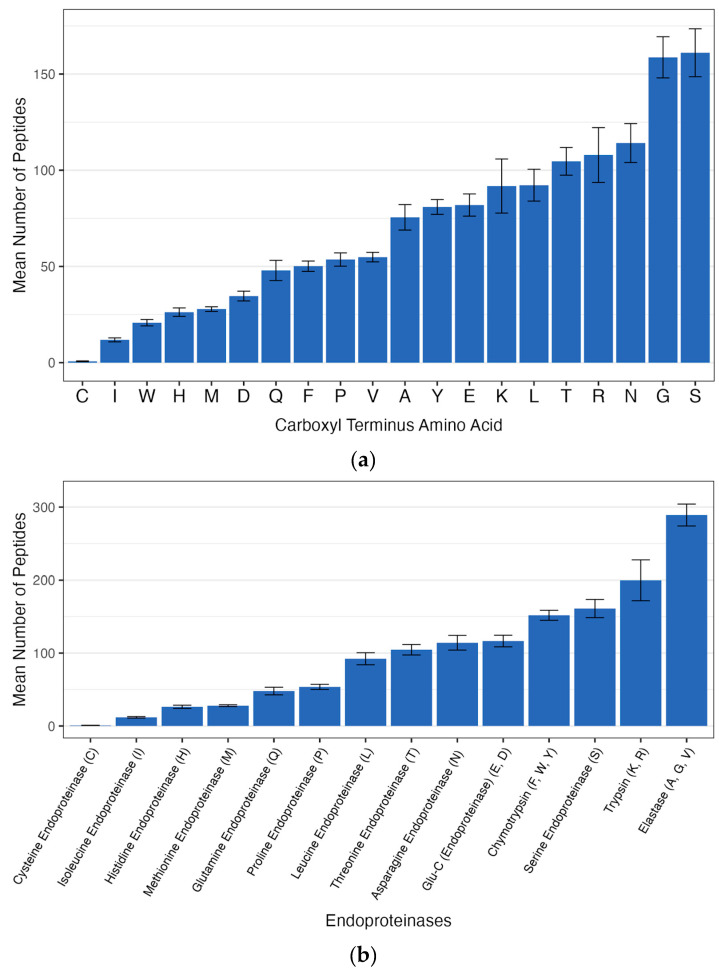
(**a**) Carboxyl terminus amino acid peptide generation in gravity-filtered aged seawater (GFASW); (**b**) Endoproteinase activity determined based on mean carboxyl terminus amino acid peptide generation in GFASW.

The most frequent carboxyl terminus amino acids in the peptides generated from the proteases in gravity-filtered aged seawater (GFASW) were serine (161 ± 45 peptides), glycine (158 ± 38 peptides), and asparagine (114 ± 36 peptides) (Figure 1). The least common carboxyl terminus amino acid present in the generated peptides was cysteine, which averaged less than one peptide per sample.

The most common endoproteinases (determined by the carboxyl terminus amino acids of generated peptides) found in the seawater samples were elastase (C-terminus amino acids A, G, and V), trypsin (C-terminus amino acids K and R), and serine-cleaving endoproteinases (S C-terminus amino acid) with means of 289 ± 54, 199 ± 100, and 161 ± 45 peptides, respectively. Eight different endoproteinases generated over 100 peptides or 100 peptides within the margin of error. Six endoproteinases generated less than 100 peptides. In total, 13 proteases were detected.

**Figure 2:**
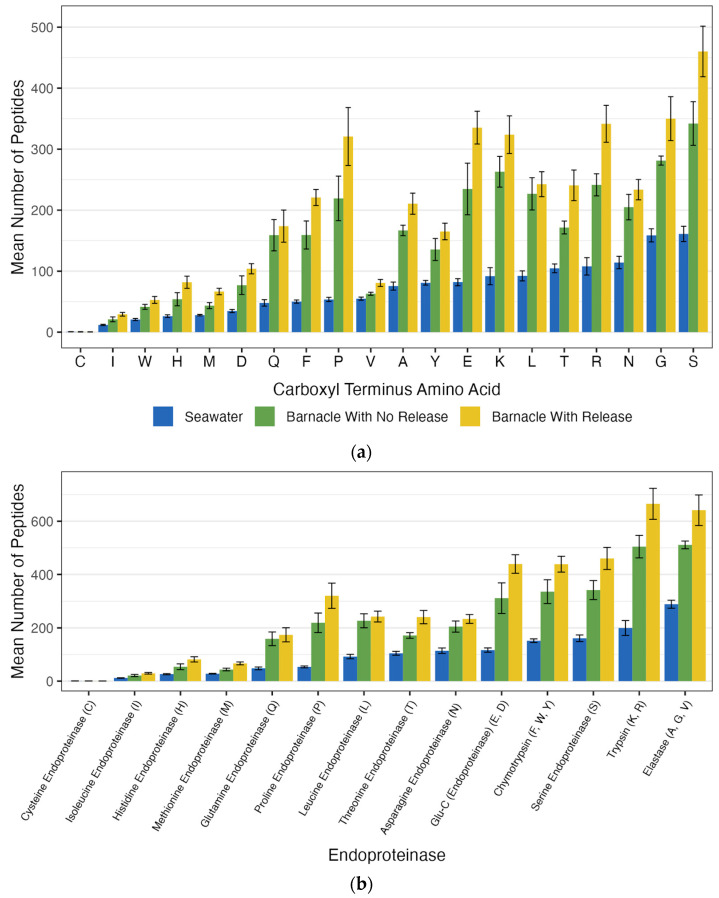
(**a**) Carboxyl terminus amino acid peptide generation after barnacle and seawater treatments. (**b**) Endoproteinase activity, determined based on carboxyl terminus amino acid peptide generation after barnacle and seawater treatments.

Barnacle treatments exhibited between 2.22 and 2.79-fold more peptide generation than seawater samples (Figure 2). The most frequent carboxyl terminus amino acids in the generated peptides from barnacles that released larvae were serine (460 ± 137 peptides), glycine (350 ± 120 peptides), and arginine (342 ± 101 peptides). The barnacles that did not release larvae had the highest number of peptides with carboxyl terminus amino acids of serine (342 ± 72 peptides), glycine (281 ± 15 peptides), and lysine (263 ± 50 peptides).

The same 13 proteases detected in seawater were also associated with barnacles. However, the patterns of activity were different. For example, seawater samples had higher percentages of glycine (11.4% versus 9.1% from non-releasing barnacles and 8.7% from releasing barnacles) and asparagine (8.2% versus 5.8% for non-releasing barnacles and 6.6% for releasing barnacles) as the carboxyl terminus amino acid in the peptides. Barnacle treatments had higher percentages of lysine (8.0% from releasing barnacles, 8.5% from non-releasing barnacles, and 6.6% in seawater), glutamic acid (8.3% from releasing barnacles, 7.6% from non-releasing barnacles, and 5.9% in seawater), and proline (8.0% from releasing barnacles, 7.1% from non-releasing barnacles, and 3.8% in seawater) as the carboxyl terminus amino acid in the peptides.

The three endoproteinases that generated the highest number of peptides in all the barnacles tested were much more active than in the seawater samples. In barnacles that released larvae, the most active endoproteinases were trypsin with 665 ± 193 peptides, elastase with 641 ± 191 peptides, and serine with 460 ± 137 peptides. In barnacles that did not release larvae, elastase (511 ± 29 peptides), trypsin (505 ± 84 peptides), and Glu-C endoproteinase (439 ± 116 peptides) generated the most peptides. In larvae-releasing barnacles, all the endoproteinases except for methionine, isoleucine, and cysteine endoproteinases generated more than 100 peptides. With the exception of histidine endoproteinase, which generated fewer than 100 peptides, barnacles that did not release larvae showed nearly identical results to those that did release larvae. Barnacles that released larvae had a 2.79-fold higher total number of generated peptides compared to seawater, whereas non-releasing barnacles had a 2.22-fold increase in total generated peptides compared to seawater.

**Figure 3:**
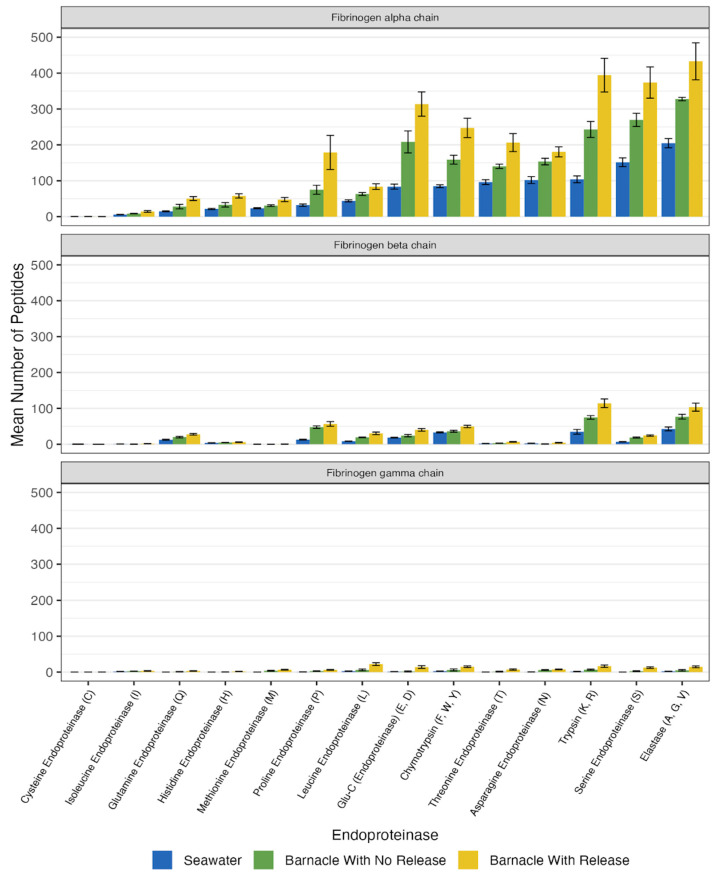
Endoproteinase activity, determined based on carboxyl terminus amino acid peptide generation by all treatments, separated by protein subunits.

The highest number of peptides were generated from the fibrinogen alpha chain across both the barnacle and seawater samples (Figure 3). The next highest number of peptides were generated by the three casein protein subunits (alpha-S1-casein, alpha-S2-casein, and beta-casein (See Supplementary Figure S1)). The fibrinogen alpha chain generated over five times the number of peptides generated by the fibrinogen beta chain and over twenty-seven times the number of peptides generated by the fibrinogen gamma chain.

## 3. Discussion

### 3.1. Endoproteinase-Generated Peptides in Seawater

The peptide products from pure proteins show a substantial background of enzymes even in gravity-filtered seawater, which enabled the detection of very low levels of free amino acids [23]. Gravity-filtered aged seawater without added pure proteins has very low levels (in the range of tens to low hundreds) of generated peptides and has less endoproteinase activity than the solutions incubating barnacles and their microbiomes. However, all 13 endoproteinases associated with barnacles and their microbiomes were also detected in gravity-filtered aged seawater.

### 3.2. Endoproteinase-Generated Peptides Based on Hatching Status

The different relative abundances of enzymes contribute to the mechanism for the recognition of organisms by their body odors. Each organism and its associated microbiome have a distinct set of enzymes and proteins (both individual- and species-specific) that contribute to its own unique odor. Interestingly, we did not observe a dramatic increase in enzyme activity with egg hatching. The increase in peptides during hatching that we did observe is likely due to the greater ventilation rates associated with larval release. This pattern could vary by species, as some barnacles brood eggs on the outside of their mantle [24]. Unique hatching mechanisms may alter ventilation rates and therefore the perceived endoproteinase abundance. The most active endoproteinases were similar across the hatching treatments. All barnacle treatments contained elastase and trypsin as part of the top three most active endoproteinases, with the third most active endoproteinase being either serine or Glu-C endoproteinase.

### 3.3. Sensitivity of Pure Proteins to the Suite of Endoproteinases

**Figure 4:**
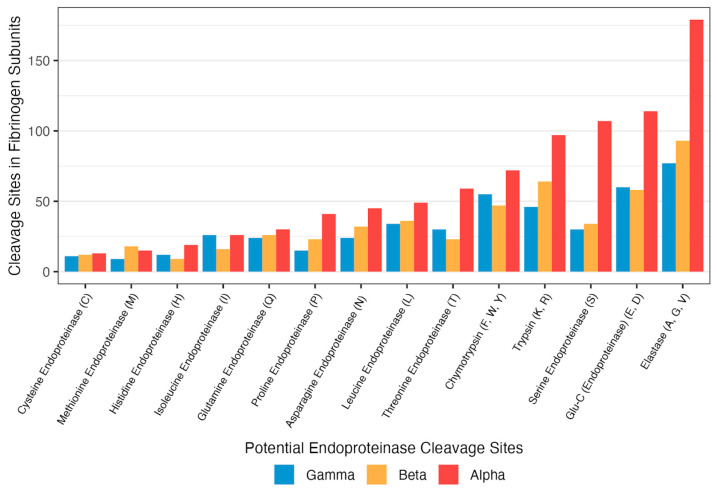
Potential endoproteinase cleavage sites in fibrinogen subprotein units based on amino acid frequency.

There are distinct impacts of different suites of enzymes on pure protein subunits, which is demonstrated by the range of peptides generated across fibrinogen alpha, beta, and gamma chains, as shown in Figure 3. Despite the subunits showing vastly different quantities of peptides, all subunits share the same pattern (Figure 2), where releasing barnacles show the highest peptide generation and seawater has the lowest. Given this information and the fact that certain chains also have greater levels of potential cleavage sites for abundant endoproteinases (Figure 4), we can conclude that the sensitivity to enzymatic digestion by the consortia of enzymes is related not only to the location of cleavage sites within the molecule, but also to the secondary structure and the frequency of specific amino acids within a helix or sheet structure. The ease and predictability of peptide generation were likely important to the evolution of peptide signaling molecules.

### 3.4. Future Studies: Understanding Exoproteinase-Generated Peptides

Here, we have organized and implemented analytical techniques to initiate a greater understanding of endoproteinase datasets. It is important to acknowledge that overlapping substrate specificities among proteinases and the structures of the proteins themselves may alter cleaving patterns. We believe that this could be addressed in future studies, though a more comprehensive understanding of a few proteinases would be essential to developing the pipelines. This would also involve more intensive data analysis.

Furthermore, exoproteinases are also represented in our proteomics data. Exoproteinases degrade endoproteinase-generated peptides one amino acid at a time from the ends of peptides. Because it depends on the number of available protein termini, exoproteinase activity is slower to develop than endoproteinase activity. With thousands of peptides generated from pure proteins in each incubation, logic coding and an abundance of time could lead to the identification of the exoproteinases present.

Because the entire sequences of the pure proteins are known, we are confident that we can sort out dominant exoproteinases by identifying gaps in peptide sequences where an amino acid or two have been removed. Future analysis will include a coding pipeline that groups similar peptide sequences that share the same string of amino acids. From there, those groupings can be sorted based on peptide length (from highest to lowest). Once the peptides are determined to be real (either by a program or by human verification), peptides from the same sample with the exact same sequence can be grouped. The relative frequency of peptides with one less amino acid than other peptides above them in the list could show the relative exoproteinase activity of the functional microbiome.

## 4. Materials and Methods

### 4.1. Water Treatments

Ambient single-pass sand-filtered seawater (pH 8.2 and 35 PSU) was used to generate filtered aged seawater (FASW) and gravity-filtered aged seawater (GFASW). FASW was generated by passing seawater through a series of filters that removed particles > 1 µM and then aging that water with aeration for at least 2 weeks in 200 L Nalgene containers [25]. GFASW was generated by gravity-filtering FASW through a sterile 0.2 µM filter (Corning 430049 0.2 µM Lot 00424001, Sigma-Aldrich, St. Louis, MO, USA) apparatus with a reservoir. The filter was pre-rinsed with 50 mL of gravity-filtered DI water, rinsed twice with FASW, and then washed by filtering with 50 mL of FASW. A 100 mL volume of FASW was then added and the filtrate was used in the following experiments.

### 4.2. Pure Proteins

Three stock solutions (2 mg/mL Ubiquitin, 1 mg/mL Casein, and 42.8 mg/mL Fibrinogen in GFASW) were used to generate solutions containing 2 µM Ubiquitin, 1 µM Casein, and 1 µM Fibrinogen in 0.2 µM filtered GFASW. Calculations were based on the subunit weight. One-milliliter aliquots of solution were frozen at −80 °C until use. Before experimentation, two aliquots for each sample were thawed, mixed thoroughly, and diluted 1 to 1 with 0.2 µM GFASW to generate incubation mixtures that were 34.37 PSU and consisted of 1 µM Ubiquitin and 0.5 µM of both Casein and Fibrinogen.

### 4.3. Experimental Design

Barnacles were collected from Beaufort, North Carolina. Larvae were mass cultured to the settlement stage [25] and settled and raised in the lab on Polydimethylsiloxane (PDMS) coatings until they reached 4 mm in basal diameter [26]. Individual barnacles were pushed off the PDMS coatings and cultured without attachment to a surface. The culturing occurred in fingerbowls with newly hatched brine shrimp for food until sexual maturity was reached, determined by the visibility of egg masses though the translucent base plate. Barnacles with mature, dark gray-colored eggs were moved to a dry fingerbowl and placed into 28 °C air overnight. When returned to water, barnacles with mature nauplii released larvae.

In order to begin the experiment, barnacles were rinsed with GFASW and blotted on a paper towel. Each barnacle was placed in an individual well in a 24-well plate (Corning Falcon 353047 24-well plate, Lot 9344002, Corning Incorporated-Life Sciences, Durham, NC, USA) at 28 °C with the lid off for experimentation until dry. Two aliquots for each barnacle were defrosted and thoroughly mixed by vortexing. At the time of experimentation, 1 mL of the pure protein solution was added to the barnacle’s well along with 1 mL of GFASW. For the control treatment, a 1 mL aliquot of the pure protein solution and 1 mL of GFASW were added to a well without a barnacle. Hatching was recorded when nauplii were seen swimming. Barnacles with hatching eggs also showed strong ventilation movements (Supplementary Video S1). After 10 min, the barnacle was removed from the treatment well and returned to a finger bowl. The solutions in the 24-well plate were incubated for 10 min and then 1.5 mL of each solution was centrifuged briefly to pellet any nauplii, transferred to new tubes, and frozen at −80 °C. In total, 15 samples were collected: 5 samples were seawater controls, 6 samples were from barnacles that released larvae, and 4 were from barnacles did not release larvae.

### 4.4. Coding Methodology

LC electrospray MS/MS data were analyzed with a protein threshold of 99%, a minimum of 1 peptide, and a 1.0 false discovery rate. Fragpipe’s protein mix database and the results from the LC-MS/MS 60 SPD method from an Orbitrap Astral were imported to RStudio (version 4.3.1, The R Foundation for Statistical Computing, Vienna, Austria). The code utilized is provided in the Supplementary Information Section. Datasets were uploaded to the RStudio file and transformed into pivot tables, allowing for a format with the spectral count in one column and treatment group in an adjoining column. The Mutate tool was used to identify the last amino acid from each peptide sequence and these amino acids were placed in a new column. Next, the Mutate tool was used to extract identifying information from the tables, including treatment type and species. Finally, the Mutate tool read the last amino acid of the sequence and created a new column in the dataset with the associated endoproteinase responsible for cutting at that amino acid. Endoproteinases were identified based on the amino acid of the C-terminus, utilizing the protease guide from the Handbook of Proteolytic Enzymes [27]. The Rbind tool was used to combine barnacle data and seawater control data into one table.

In our analysis, any spectral count above 0 was considered “present” (0 = not present, 1 = present). This process required the Mutate tool to turn any spectral count greater than 1 to 1. The peptide sequences were grouped by individual treatment label, treatment group, and the last amino acid. The number of unique peptide sequences in each of these samples was then totaled. Mean and variance were calculated for the generated peptides based on the carboxyl terminus amino acids and separately for the associated endoproteinases. All graphs were created using ggplot2 (version 3.4.4, Hadley Wickham, New York, USA) and all error bars are the standard error of the mean.

## 5. Conclusions

This project identified endoproteinases based on the peptides they produced from known pure proteins. All treatments contained 13 broad groups of endoproteinases. The top two enzymes found (elastase and trypsin) are the most common and most ancient of all the known proteases. A more detailed examination of the data should result in the identification of all known endoproteinases associated with some organisms and their microbiomes. There were distinct patterns and frequency differences between barnacles and their associated microbiomes and seawater samples. The enzyme patterns for barnacles are similar at all reproductive states, including larval release, where sequences of known mimics of pheromones and signals dominate. The easiest pure protein subunit to degrade was the fibrinogen alpha chain, suggesting that the position in the molecule, secondary structure, and amino acid frequencies are all important when proteins are recycled.

We demonstrate that we can describe the endoproteinases in functional host/microbiome communities using pure protein incubations with LC electrospray MS/MS proteomic analysis of the resulting peptides to ascertain the dominant endoproteinases. These concepts can be applied to other degradative enzymes and metabolomics as technology develops. Future studies will explore exoproteinases and their impact on endoproteinase analysis.

## Data Availability

The original data presented in the study are openly available in the Box folder at https://duke.box.com/s/7ggbyc1ed9c1kn3y29fhy38ww8y1tpzo, accessed on 17 October 2025.

## Supplementary Materials

The supporting information can be downloaded at: https://www.mdpi.com/article/10.3390/ijms262311393/s1.

## Abbreviations

The following abbreviations are used in this manuscript:

FASW	filtered aged seawater
GFASW	gravity-filtered aged seawater
PDMS	polydimethylsiloxane
