# Supplementary figures and images for: Egg Hatching, Peptide Pheromones, and Endoproteinases in Barnacles
Source: Int J Mol Sci. 2025 Nov 25;26(23):11393. doi: 10.3390/ijms262311393 (PMC12691806; doi:10.3390/ijms262311393)

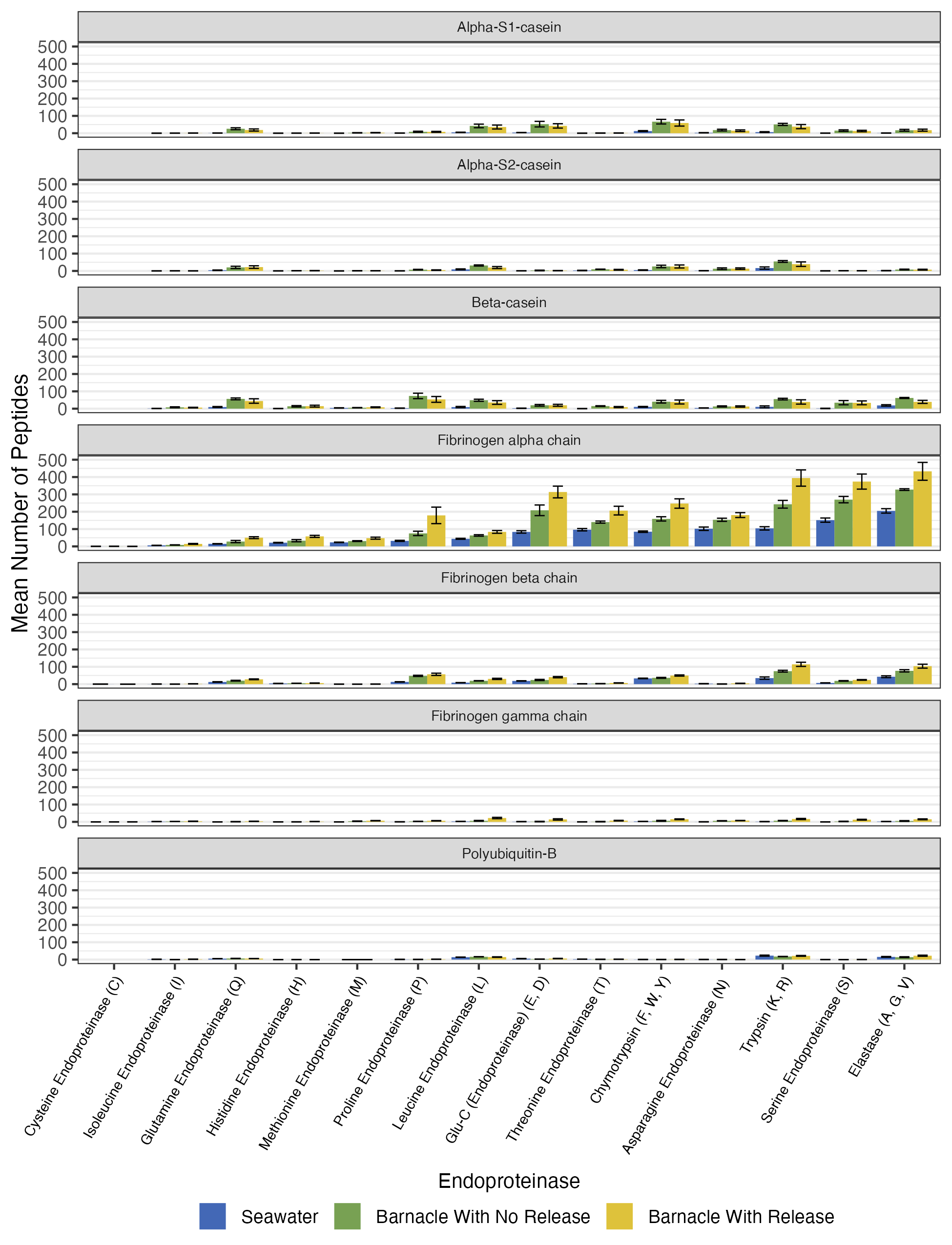

Supplement: Supplementary file 1 [file ijms-26-11393-s001.zip › Barnacle Endoproteinase Supplement/Supplementary Videos and Photos/Figure S1. AllPureProteinEndoprot.png]
